# Diaphragm Ultrasound in Different Clinical Scenarios: A Review with a Focus on Older Patients

**DOI:** 10.3390/geriatrics9030070

**Published:** 2024-05-30

**Authors:** Carmine Siniscalchi, Antonio Nouvenne, Nicoletta Cerundolo, Tiziana Meschi, Andrea Ticinesi, on behalf of the Parma Post-Graduate Specialization School in Emergency-Urgency Medicine Interest Group on Thoracic Ultrasound

**Affiliations:** 1 Department of Continuity of Care and Multicomplexity, Azienda Ospedaliero-Universitaria di Parma, Via Antonio Gramsci 14, 43126 Parma, Italy; csiniscalchi@ao.pr.it (C.S.); antonio.nouvenne@unipr.it (A.N.); ncerundolo@ao.pr.it (N.C.); tiziana.meschi@unipr.it (T.M.); 2 Department of Medicine and Surgery, University of Parma, Via Antonio Gramsci 14, 43126 Parma, Italy

**Keywords:** ultrasound imaging, hospitalized patients, respiratory failure, diaphragm dysfunction, sarcopenia, heart failure

## Abstract

Diaphragm muscle dysfunction is increasingly recognized as a fundamental marker of several age-related diseases and conditions including chronic obstructive pulmonary disease, heart failure and critical illness with respiratory failure. In older individuals with physical frailty and sarcopenia, the loss of muscle mass and function may also involve the diaphragm, contributing to respiratory dysfunction. Ultrasound has recently emerged as a feasible and reliable strategy to visualize diaphragm structure and function. In particular, it can help to predict the timing of extubation in patients undergoing mechanical ventilation in intensive care units (ICUs). Ultrasonographic evaluation of diaphragmatic function is relatively cheap, safe and quick and can provide useful information for real-time monitoring of respiratory function. In this review, we aim to present the current state of scientific evidence on the usefulness of ultrasound in the assessment of diaphragm dysfunction in different clinical settings, with a particular focus on older patients. We highlight the importance of the qualitative information gathered by ultrasound to assess the integrity, excursion, thickness and thickening of the diaphragm. The implementation of bedside diaphragm ultrasound could be useful for improving the quality and appropriateness of care, especially in older subjects with sarcopenia who experience acute respiratory failure, not only in the ICU setting.

## 1. Introduction

The diaphragm is the musculotendinous anatomic barrier between the thoracic and abdominal cavity. It plays a crucial role in respiratory homeostasis; injury to the diaphragm impairs ventilation and oxygen exchanges [1]. In addition to the respiratory functions, it contributes to non-respiratory activities, such as sternutation, vocalization, swallowing, as well as emesis, urination and defecation by increasing intra-abdominal pressure and prevention of gastroesophageal reflux by exerting external pressure at the esophageal hiatus [2]. Conditions that interfere with the regular operations of the diaphragm, such as muscle wasting, chronic obstructive pulmonary disorder, heart failure, neuromuscular disease, critical illness, tumor, medications and metabolic abnormalities can result in diaphragmatic dysfunction and, at the same time, diaphragmatic dysfunction can stratify the severity of these conditions [3,4]. 

A number of static and dynamic imaging techniques are used in the evaluation of patients suspected of diaphragm dysfunction [2]. Static imaging techniques are used to assess the position, shape and dimensions of the diaphragm and include chest radiography [5], computed tomography (CT) [6] and static magnetic resonance imaging (MRI) [7]. Dynamic imaging techniques are used to assess diaphragm motion in one or more directions. This group of imaging techniques includes fluoroscopy [8] and dynamic MRI [9]. 

In the last decade, ultrasound has also emerged as a reliable and reproducible technique to assess diaphragm structure and function both statically, thanks to brightness B-mode techniques [10,11], and dynamically, using motion M-mode ultrasonography [12]. The purpose of this review is to evaluate the clinical usefulness of ultrasound evaluation of the diaphragm muscle in different clinical settings, with a particular focus on older subjects, who are more prone to diaphragm dysfunction due to the physiological process of aging of the respiratory system, and due to the high prevalence of physical frailty and sarcopenia. 

## 2. Methods

We performed a literature search on PubMed and Scopus as of February 20th, 2024, using a search strategy combining one of the following terms, “diaphragm ultrasound”, “diaphragm dysfunction”, “diaphragm excursion”, “diaphragm thickness”, with one of the following terms related to the application of ultrasound technique: “geriatric patients”, “older patients”, “sarcopenia”, “acute sarcopenia”, “respiratory sarcopenia”, “frail older patients”, “intensive care unit”, “mechanical ventilation”, “non-invasive ventilation”, “respiratory failure”, “weaning failure”, “COPD”, “COVID-19”, “pneumonia”, “congestive heart failure”, “paralysis”, “stroke”, “lateral amyotrophic sclerosis”, “acute care”, “outpatient clinic”. The literature search and first selection of papers were performed by junior authors, who also checked the results, after the removal of duplicates, and considered them for inclusion in this review based on their relevance for the study aims. Supervising senior authors (C.S., T.M. and A.T.) subsequently checked the selected papers, and chose for inclusion in the review those with particular relevance for the clinical development of diaphragm ultrasound and those with particular importance for the care of geriatric patients. Only papers published in the English language were considered. The results are presented in narrative form, since a high heterogeneity of clinical settings, characteristics of studied populations, ultrasonographic methodology and definitions of diaphragmatic dysfunction were identified across the literature. 

## 3. Ultrasound Imaging of the Diaphragm

### 3.1. General Historical Background

Diaphragm ultrasonography was first used in the late 1960s to determine the position, size and anatomical relationships of supra- and subphrenic mass lesions [13]. Two decades later, Wait et al. developed a technique to measure diaphragm thickness based on ultrasonography [14]. Since those seminal works, investigators have published a growing number of studies on the use of ultrasonography to evaluate the diaphragm’s thickness, strength and recruitment during voluntary contractions [15,16,17], but the technique was practiced only by a niche group of experts.

The interest in diaphragm ultrasound was revived from the late 2000s onwards, especially in an intensive care unit (ICU) setting. Diaphragmatic dysfunction, in fact, is particularly frequent in critical illness, and its assessment may have fundamental prognostic implications [17]. The measurement of diaphragm inspiratory excursion, thickness and thickening fraction during mechanical ventilation can, in fact, be predictive of the optimal timing of weaning from mechanical ventilation and its success [17]. 

Apart from the ICU setting, studies on diaphragm ultrasound in acute and chronic cardiorespiratory illnesses are less numerous, though constantly increasing in number in recent years. This technique is not part of daily clinical practice in medical wards yet. 

However, the growing interest towards age-related sarcopenia, i.e., the loss of muscle mass and function frequently seen in older patients and responsible for a wide range of adverse outcomes including disability and mortality [18], has been accompanied by particular attention applied to the role of musculoskeletal imaging in the geriatric setting. Although peculiar in anatomical and physiological terms, the diaphragm is part of the musculoskeletal system and is affected by sarcopenia exactly like the muscles of other body districts. 

The coexistence of respiratory muscle weakness and reduced respiratory muscle mass has recently been defined as a separate entity called “respiratory sarcopenia” [19]. Ultrasound has, thus, been recognized as a technique of potential clinical usefulness in the assessment of this condition, as part of routine geriatric assessment [19]. Ultrasonographic assessment of lower limb muscles, in fact, has proven effective as a screening method for sarcopenia in older people [20], and recognized as such also by the European Working Group on Sarcopenia in Older People (EWGSOP) [18]. By analogy, diaphragm ultrasound may represent a promising method of assessment of respiratory sarcopenia not only in geriatric patients but also in patients who experience acute muscle wasting due to an acute illness, and this is perhaps the most innovative application of this technique. 

### 3.2. Technique of Diaphragm Assessment by Ultrasound

According to a recent methodological systematic review and meta-analysis of the published literature, diaphragm ultrasound is a valid, reliable and reproducible tool to assess diaphragmatic dysfunction and monitor its evolution in ICU patients [21]. However, significant discrepancies in the technique of assessment exist across studies, especially in the earlier ones [21]. The current literature, anyway, allows us to recommend an assessment protocol based on the best practices identified across the published studies [22]. The following description of how bedside diaphragm ultrasound can be performed is based on methodological papers retrieved from the scientific literature and expert recommendations [22,23,24,25,26,27,28,29]. All the pieces of equipment and software required to perform this examination are generally available in typical ultrasound machines used at the patient’s bedside in hospital settings. Standard ultra-portable wireless devices, that are increasingly used for performing ultrasonography in the community setting, may instead lack, in some cases, the required software. 

**Figure 1:**
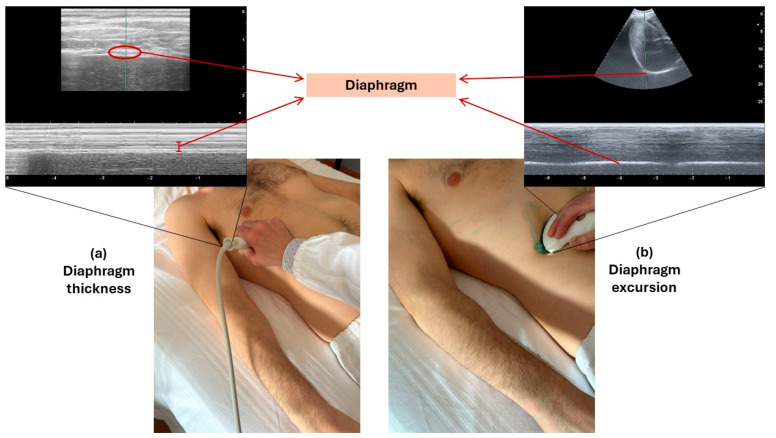
Ultrasound scans required for measuring diaphragm thickness (**a**) and diaphragm excursion (**b**). The linear ultrasound probe should be first put in the right eighth, ninth or tenth intercostal space, with an oblique or transversal scan, in order to visualize the zone of apposition as the area where the curtain sign appears under quiet inspiration. The diaphragm can be visualized as the structure between two parallel hyperechoic lines just below the curtain sign (**a**). Then, the convex probe should be put in the right subcostal region, directing the probe upwards in order to exploit the acoustic window of the liver. The diaphragm appears as the cupola-like hyperechoic structure surrounding the right liver lobe, exhibiting movements synchronous with respiration (**b**). Both diaphragm thickness and excursion need to be measured with the M-mode function of the ultrasound turned on, asking the patient to perform quiet breathing and maximal voluntary inspirations.

**Figure 2:**
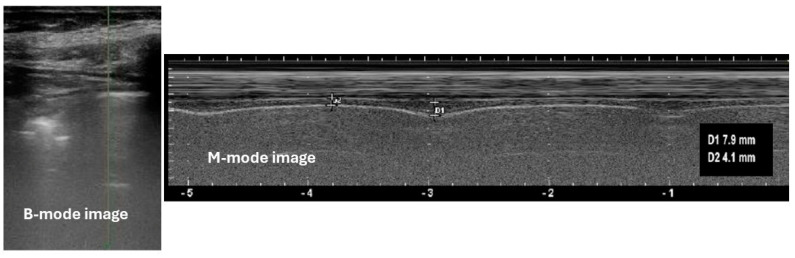
Ultrasonographic appearance of diaphragm thickness in an older subject with heart failure during quiet breathing. The diaphragm is visualized in the B-mode image as the track-like structure lying between two hyperechoic parallel lines. Measures of thickness during inspiration and expiration on quiet breathing are taken after activating the M-mode ultrasound function. The distance between the two parallel hyperechoic lines appears to increase in synchrony with inspiration (measure of thickness 7.9 mm) and to decrease during expiration (measure of thickness 4.1 mm).

Although ultrasound can theoretically allow us to visualize also the left hemidiaphragmatic cupola, basic assessment is limited to the right hemidiaphragm, exploiting the acoustic window offered by the liver. The patient must rest in a semi-recumbent position, on his/her back. The right eighth, ninth or tenth intercostal spaces must be identified by palpation on the area between the anterior and the mid-axillary line. A 5–12 MHz linear ultrasound probe must be put in these intercostal spaces, with an abundant amount of gel, perpendicular to the chest wall, to identify the diaphragm zone of apposition (ZOA) with B-mode imaging (Figure 1). The ZOA is the chest wall area where the lower rib cage reaches the abdominal content. During inspiration, diaphragm contraction and lung inflation make this area of the chest wall in contact with lower regions of the lung parenchyma [30]. Ultrasonographically, the ZOA is visualized with the so-called “curtain sign”, representing the descending movement of lung parenchyma following inflation due to diaphragm contraction [30]. The diaphragm can be identified next to the ZOA as the structure lying between two hyperechoic parallel lines, representing the pleural and peritoneal lines generated at the acoustic interface between the corresponding organ parenchyma and the barrier structure (Figure 2) [31]. 

The M-mode function must be then turned on. The dynamic changes of diaphragm thickness on the reference plane will be then visualized during respiratory cycles (Figure 2). Image freezing will then allow examiners to measure thickness during different timings of the respiratory cycle. First, the patient should be asked to breathe quietly, and diaphragm thickness should be measured on end-inspiration, corresponding to tidal volume (TV), and end-expiration, corresponding to functional residual capacity (FRC). Then, the patient should be asked to perform maximal voluntary inspirations to total lung capacity (TLC). Diaphragm thickness should be measured on end-inspiration also in this case, to obtain the maximal thickness corresponding to the maximal voluntary contraction of the muscle. The diaphragm thickening fraction (Tdi) on tidal volume (diaphragm thickness on TV minus diaphragm thickness on FRC) and on peak inspiration (diaphragm thickness on TLC minus diaphragm thickness on FRC) can then be calculated. Repeated measures are generally required to assure reliability. Methodological investigations conducted in different settings, including in the ICU and with healthy volunteers, suggest that this protocol is reproducible and repeatable, and the obtained measures are significantly correlated with spirometric parameters [23,24,25,26].

**Figure 3:**
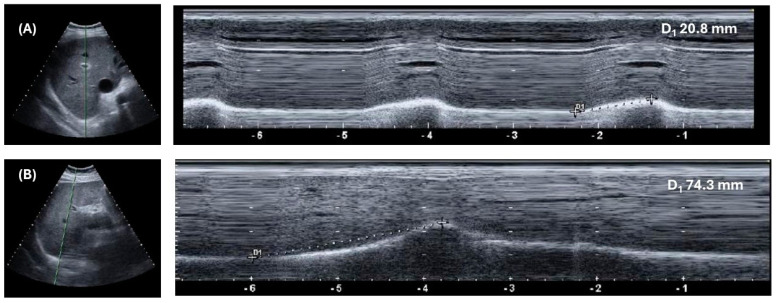
Ultrasonographic measurement of diaphragm excursion in an older subject with heart failure on quiet breathing (**A**) and maximal voluntary inspiration (**B**). The diaphragm is visualized in the B-mode subcostal scan as the hyperechoic cupola-like line surrounding the liver parenchyma. The movements of this line on quiet breathing (**A**) and maximal voluntary inspiration (**B**) are then assessed activating the M-mode ultrasound functionality, which allows us to visualize peaks in the hyperechoic line correspondent to the diaphragm, synchronous with breaths. The height of these peaks corresponds to diaphragm excursion.

Diaphragm excursion can be also easily assessed by ultrasound [27]. In this case, a 3.5–5 MHz convex probe must be used, to allow deeper penetration of ultrasound waves. The probe should be put in the right hypochondrium, between the midclavicular and anterior axillary lines, immediately under the costal margin, and the ultrasound waves must be pointed upwards, in order to visualize the right diaphragm hemicupola in the lower part of the image, below the liver parenchyma (Figure 1). The M-mode function must be then turned on. Diaphragm movements can be visualized, in synchrony with breathing cycles, as a sinusoid movement of the hyperechoic line representing the interface between the abdominal and chest cavities. Image freezing will allow examiners to measure the amplitude of diaphragmatic excursions on quiet breathing (i.e., tidal volume) and on maximal voluntary inspiration, asking for the collaboration of the patient in performing such respiratory acts (Figure 3). Although these measures are generally easier to obtain than those of thickness, repeated assessment is recommended to assure reliability. This technique is also reproducible and related to measures of diaphragmatic excursions obtained with traditional radiographic techniques [27,28]. 

Ultrasound evaluation of diaphragm motility can be obtained also indirectly, by measuring the craniocaudal displacement of the left branch of the portal vein during breathing [29]. However, this method is not common in clinical practice and research. 

The assessment can also be completed with the measurement of the length of the diaphragm ZOA during maximal inspiration, which is well correlated with pulmonary volumes [31]. Although this parameter can provide clinically useful information, avoiding the need for performing spirometry, it is not part of the usual ultrasound evaluation of the diaphragm. 

Finally, some research groups have proposed to integrate ultrasound imaging of the diaphragm with shear-wave elastosonography [32]. This is an ultrasound technique providing non-invasive assessment of tissue elasticity, based on the emission of high-energy acoustic pulses from the ultrasound probe, inducing compression in body tissues that produce, as a reaction, shear waves detected and analyzed by the probe [33]. Elastosonography has been mainly used for detecting and measuring fibrosis in solid parenchymatous organs, such as the liver or the thyroid [33]. However, it has also been applied to the study of the elastic properties of the lung parenchyma in chronic obstructive pulmonary disease (COPD) [34]. 

Two recent studies suggest that diaphragm stiffness estimated by ultrasonography is significantly correlated with pulmonary volumes and spirometric parameters, such as the Tiffeneau index, in COPD, predicting the number of acute exacerbations over time and stratifying disease severity [35,36]. In critically ill patients undergoing mechanical ventilation, diaphragm stiffness estimated by shear-wave elastosonography was correlated with transdiaphragmatic pressure, providing useful information for estimating respiratory load and regulating ventilatory settings [37,38].

### 3.3. Reference Values of Diaphragm Ultrasound Parameters

The normative values of diaphragm excursion and thickness in adult patients were first established by the pioneering studies by Boussuges et al. [27] and Ueki et al. [39], respectively. Diaphragm excursion on quiet breathing was considered normal above 9 mm in females and 10 mm in males, while the reference values for deep breathing were considered above 37 mm in females and 47 mm in males [27]. Ueki et al. assessed diaphragm thickness only in male subjects, finding an average thickness of 1.7 ± 0.2 mm on FRC (end-expiration) and 4.4 ± 1.4 mm on TLC (maximal voluntary inspiration) [39]. Since then, other research groups have analyzed diaphragm ultrasound in healthy individuals, in order to establish reference values for adults [24,25,40,41,42,43]. The findings are summarized in Table 1. Recently, reference values have been established also in the pediatric population, where diaphragm ultrasound is increasingly used for clinical indications similar to the ones of adult and older patients [44].

The normative values of diaphragm excursion, both on quiet breathing and maximal voluntary inspiration, were significantly lower in females than in males [25,40]. In a large group of subjects from Egypt, Kabil et al. also found a significant trend towards reduction of diaphragm excursion on quiet breathing across increasing age categories, but the excursion on deep breathing was surprisingly increased over the age of 65 years old [40]. Diaphragm excursion was also affected by body mass index (BMI), with a trend towards increases on quiet breathing and decreases on deep breathing in subjects with overweight and obesity [40]. The available studies establishing normative values, however, suffer from reduced sample sizes and heterogeneity of the ethnic provenience of participants. 

Despite this evidence, however, there is no consensus agreement on the optimal cut-offs for defining diaphragm dysfunction. A recent systematic review of the studies investigating the predictive capacity of diaphragm ultrasound for weaning from mechanical ventilation in the ICU setting highlighted a high level of heterogeneity of the cut-offs used for defining diaphragm dysfunction across studies [45]. A diaphragm excursion of <1 cm on quiet breathing, however, is generally considered pathologic [45].

Diaphragm thickness, instead, seems unaffected by age, sex or body habitus [41,42]. Only the study by Carrillo-Esper et al. found significant differences in thickness between men and women [43]. The variation in thickness during tidal volume (Tdi) is considered normal when above 20%, even though a significant portion of normal individuals show negligible or no diaphragm thickness variation on quiet breathing [41,42].

The studies, whose findings are summarized in Table 1, also suffer from a significant degree of heterogeneity in ultrasound procedures, and, in many cases, from small sample sizes. Although expert recommendations exist, to date there is no consensus on a universal protocol of diaphragm ultrasound assessment. Therefore, in this context, the establishment of normative values for diaphragm thickness, thickening fraction and excursion in ultrasound is challenging. The gold standard technique for assessing diaphragm excursion remains dynamic MRI, which is ideal for research protocols but not feasible in clinical practice. Interestingly, the dynamic MRI cut-offs for normal diaphragm excursion on deep breathing are lower (44 ± 4 mm) than those detected in ultrasound studies summarized in Table 1 [46].

Recent studies have also clarified the normal values of diaphragm thickness, thickening ratio and excursion under particular circumstances, such as in children under 8 years old [47], during sniff maneuvers [48] and in the seated position [49,50]. Sniff maneuvers, i.e., forced expiration through occluded nostrils, can be useful in assessing diaphragmatic dysfunction in subjects with neuromuscular illnesses [48]. Diaphragm ultrasound can also be performed with the patient in the seated position in cases of phrenic nerve paresis [49,50]. No significant sex differences were detected either in these studies, while age-related variations were not investigated.

### 3.4. Advantages and Disadvantages of Diaphragm Ultrasound

Ultrasound assessment of diaphragm thickness and function is generally considered safe, feasible and accurate [51,52]. Basic ultrasound equipment, with a linear 5–12 MHz and a convex 3.5–5 MHz probe, is sufficient to perform high-quality examinations [53]. No ionizing radiations are involved, and no invasive maneuvers are required [53]. The complete assessment can be performed also in a busy clinical setting, requiring a maximum of 15 min and usually less than 10 min in experienced hands [53]. The possibility of assessing diaphragm structure and function directly at the patient’s bedside, without the need for transportation to specialized services, and the possibility of integrating images and measures with the clinical history of the patient represent the most important strengths of the technique [50,51]. These advantages are particularly evident in older patients, who often suffer from mobility limitations and cognitive impairment, with a lack of collaboration [54]. 

The reproducibility of diaphragm ultrasound measures of excursion and thickness is generally considered good, with interobserver agreement correlation coefficients ranging from 0.56 to 0.989 [51,53,55], even if a certain degree of dependency on operator skills is unavoidable. The accuracy of repeated measures performed by the same operator is, however, very high, assuring the possibility of performing follow-up examinations [51,53,55]. 

Limitations of the technique include difficulties in visualizing the diaphragm, especially the left hemicupola, in obese subjects and in subjects with abdominal diseases increasing intra-abdominal pressure and prompting diaphragm upward displacement [12,56]. A certain degree of patient collaboration is also necessary, since thickness and excursion need to be measured both on quiet and deep breathing [51,52]. Acute illnesses and symptoms like cough may also limit the capacity of performing voluntary respiratory efforts even in collaborative patients [46]. All these issues may be particularly emphasized in older patients with frailty and multimorbidity, who are hospitalized with acute respiratory conditions [57]. Adequate training of operators is also an issue. The measurement of diaphragm excursion can be considered an easy task and has a steep learning curve, while evaluation of thickness is far more difficult and requires longer training [58]. 

## 4. Diaphragm Ultrasound in Specific Clinical Situations or Diseases

### 4.1. Respiratory Failure Requiring Ventilatory Support

Diaphragm dysfunction is a highly prevalent condition in patients admitted to the ICU and requiring ventilatory support for respiratory failure [59]. It is particularly pronounced in the context of severe sepsis, representing a manifestation of organ failure [59]. Ultrasonographic assessment of this condition has been particularly studied in relation to its capacity to predict the optimal timing and outcome of extubation, when performed repeatedly during the ICU stay [17]. Several systematic reviews and meta-analyses suggest that ultrasound assessment of the diaphragm thickening fraction during assisted ventilatory cycles can be helpful in predicting the outcome of weaning, while the evaluation of diaphragmatic excursion has little clinical significance in this context [17,60,61,62,63]. The reduction in diaphragm thickness, assessed ultrasonographically during an ICU stay in ventilated patients, is also significantly associated with a prolonged duration of ventilation and ICU stay, in comparison with patients whose thickness remains unchanged or improves [64]. The integration of clinical and laboratory data with diaphragm, lung and heart point-of-care ultrasonography may also improve the accuracy of the prediction, and, thus, be of great clinical importance [61]. Recently, diaphragm ultrasound has emerged as a reliable tool for monitoring respiratory function even in the pediatric population, predicting the outcome of ventilation weaning in children with critical illness [65]. 

However, pitfalls and limitations should be carefully considered. Diaphragmatic dysfunction, assessed by ultrasound, has a poor correlation with ICU-acquired weakness [66], a frequent complication of an ICU stay combining myopathy and neuropathy [67]. The cut-offs used for defining diaphragm dysfunction also show substantial variations across different studies [17]. In addition, one of the largest studies to date, conducted on 191 patients undergoing mechanical ventilation, showed no significant differences in diaphragm thickening ratio and excursion between extubation successes and failures [68]. Ventilator weaning failure, in fact, has a complex pathophysiology that frequently involves concomitant conditions that affect the heart, the lungs and other respiratory muscles as well as the diaphragm. Therefore, factors not directly affecting the diaphragm thickening ratio may be involved [69,70]. 

Few diaphragm ultrasound studies conducted in an ICU setting have been specifically focused on older individuals. In 2015, Sarwal and colleagues [71] reported the case of an 88-year-old woman with COPD, hospitalized for ischemic stroke resulting in acute hemiplegia, complicated by the onset of acute respiratory failure necessitating intubation and mechanical ventilation. Despite the intensive support and adequate sedation, persistent patient–ventilator dyssynchrony arose. Diaphragm ultrasound demonstrated passive paradoxical movements, an indirect sign of phrenic nerve paralysis resulting from ischemic disease. This case is paradigmatic of the kind of clinical information provided by point-of-care diaphragm ultrasound in older multimorbid patients in an ICU setting, going beyond the simple prediction of weaning failure. 

Overall, three studies evaluated the capacity of diaphragm ultrasound to predict weaning from mechanical ventilation, either invasive or non-invasive, in older individuals [72,73,74]. The results are summarized in Table 2, and speak in favor of the clinical application of this point-of-care method for deciding the timing of weaning and predicting the outcomes. In one study [74], diaphragm ultrasound was non-inferior to the rapid shallow breathing test, which represents the most commonly used index to predict weaning [75,76]. 

Muscle wasting is a common complication of hospitalization in geriatric patients, and is often associated with a decline in muscle function in the so-called “acute sarcopenia” syndrome [77,78]. Muscle wasting is also commonplace in adult patients admitted to the ICU [79,80], and shows a correlation with diaphragmatic dysfunction assessed by ultrasound [81]. Thus, physical frailty and sarcopenia should represent highly prevalent conditions in older patients undergoing intensive care support [82], although studies on this topic are scarce. The results highlighted in Table 2 suggest that the sensitivity of ultrasound in the diagnosis of diaphragm dysfunction is higher in older patients undergoing ventilatory support, either invasive or non-invasive, than in adult subjects. Interestingly, the diaphragm ultrasound parameter more associated with ventilation weaning outcome, in older patients, was excursion under spontaneous breathing, and not thickness or thickening ratio, as found in adult subjects. These results could be influenced by the acute sarcopenia phenomenon, involving not only skeletal muscles but also the diaphragm structure and function, which may be more pronounced in older subjects with frailty [19]. 

### 4.2. COPD 

Diaphragm dysfunction has been frequently demonstrated in patients with COPD. In this condition, the diaphragm suffers from a mechanical disadvantage, that is caused by hyperinflation of the lungs, resulting in shortening of muscular fibers and reduced contractile effectiveness [83]. This phenotype, however, is not common to all subjects. In fact, obesity, representing one of the main comorbidities of COPD significantly impairing lung function, seems to be protective against diaphragm dysfunction [84,85]. In an ultrasound study conducted on 48 stable COPD patients, the size of the diaphragm ring of insertion was positively correlated with BMI, resulting in normal diaphragm thickness and excursion in obese subjects [84]. 

Diaphragm ultrasound has been studied mainly in stable COPD patients, where its parameters show significant correlations with respiratory function tests and may be helpful in stratifying the severity of the disease [86,87,88]. In particular, a recent systematic review has highlighted that high-quality evidence from the literature supports the presence of a positive correlation between diaphragm excursion, assessed by ultrasound, and forced expiratory volume in the first second (FEV1) or forced vital capacity (FVC), assessed by spirometry [88]. Reduced diaphragm mobility, both on quiet breathing and on maximal voluntary inspiration, is, in fact, an expression of hyperinflation, and shows correlations with pulmonary volumes and the Tiffeneau index (FEV1/FVC) [89,90,91,92]. In one study conducted on 37 stable COPD patients, diaphragm mobility on quiet breathing was also negatively correlated with P_a_CO_2_ in arterial blood gas analysis [92]. 

The correlation between diaphragm excursion and pulmonary volumes is so evident that some authors have proposed to use diaphragm ultrasound as a proxy for spirometry or as an auxiliary method to interpret spirometry findings in stable COPD patients [93,94,95]. In particular, the ratio between the forced expiratory diaphragmatic excursion in the first second and maximum expiratory diaphragmatic excursion is predictive of an obstructive spirometric pattern [93]. Furthermore, diaphragmatic excursion of <67 mm at forced breathing is predictive of obstruction on spirometric tests, while normal values at this ultrasound test do not help to assess obstructive pattern and severity [94]. A phenotype characterized by low maximal inspiratory pressure on spirometry and reduced diaphragm excursion on forced breathing is particularly frequent in patients over 70 years old, is associated with impaired physical performance and could help identify subjects with respiratory sarcopenia [95]. 

From a clinical perspective, reduced diaphragm excursion assessed by ultrasound is also able to predict the performance on a 6 min walking test [94], exercise capacity and tolerance [96,97] and, most importantly, the number of yearly acute exacerbations [98].

The clinical value of assessing diaphragm thickness and thickening fraction in COPD patients is, instead, more controversial. A study conducted on 140 stable COPD patients showed that diaphragm excursion is negatively correlated with thickness and positively correlated with the thickening ratio [99]. In COPD patients, an increase in diaphragm thickening ratio during quiet breathing could be the expression of increased workload with reduced force reserve, and, thus, should be considered a marker of disease severity [100]. The thickening ratio, however, is reduced on maximal inspiration, when compared with healthy subjects [101], and the evaluation of this parameter could be helpful for stratifying the severity of COPD [102]. In fact, one study conducted on 28 older males with COPD has suggested that the thickening ratio of the diaphragm on maximal inspiration could be correlated with nocturnal peripheral oxygen saturation [103]. In spite of this, two different studies found no differences in diaphragm thickness and thickening ratio when comparing patients with COPD and healthy subjects [104,105]. 

Only a few studies evaluated diaphragmatic ultrasound during acute exacerbations of COPD (Table 3), and they were mainly conducted on severe forms with acute respiratory failure [106,107,108,109]. Subjects with acute exacerbations had lower diaphragm thickening fractions and excursions on maximal voluntary inspiration than patients with COPD but no sign of acute exacerbation [106]. According to a small study conducted in a non-intensive setting, diaphragm thickening fractions improved during the clinical course of the acute exacerbation, but not excursions [107]. In subjects necessitating non-invasive ventilation (NIV) support, a favorable clinical course was predicted by better measures of diaphragm excursion, not by thickness or its variations [108]. However, in another study conducted on 41 patients with acute exacerbation of COPD and respiratory acidosis, a diaphragm thickening fraction on quiet spontaneous breathing of <20% was associated with NIV failure, the need for mechanical ventilation and a longer ICU stay [109]. 

Overall, the data from the literature suggest that the significance of performing diaphragm ultrasound in patients with COPD may be different under stable conditions and during acute exacerbations. In chronic patients, assessment of diaphragm excursion may be very useful for stratifying the severity of the disease, avoiding repeated spirometric examinations and completing the physiological assessment, also in order to modulate treatment. In this context, diaphragm ultrasound could also represent a useful tool for monitoring the response to inspiratory muscle training [97,110,111]. During acute exacerbations, on the other side, the evaluation of diaphragm thickness and thickening fraction could be useful to guide treatment, verify response to NIV and, eventually, decide to escalate oxygen and ventilatory support. In both cases, the presence of physical frailty and sarcopenia, which is a very common comorbidity in COPD [112], may contribute to worsening diaphragm function, reducing the response to treatment and negatively affecting prognosis [19]. 

### 4.3. COVID-19, Other Pneumonia and Related Conditions 

The pandemic of coronavirus disease19 (COVID-19) has substantially contributed to the spread of the use of point-of-care thoracic ultrasonography, also in clinical contexts where it was previously neglected. In fact, the capacity of lung ultrasound to detect lung parenchymal abnormalities associated with interstitial pneumonia and their extent [113] guaranteed the application of ultrasound also in busy clinical settings, such as emergency departments [114], or in low-resource settings, including nursing homes [115]. 

Diaphragm ultrasound was also particularly studied in patients hospitalized with severe forms of COVID-19 requiring semi-intensive or intensive care support. In this context, SARS-CoV-2 infection is associated with up- and downregulation of several genes in the diaphragm muscular fibers, resulting in the activation of pathological pathways leading to fibrosis [116]. Thus, diaphragm dysfunction was hypothesized as one of the central pathophysiological mechanisms leading to severe respiratory failure in COVID-19 [117]. Interestingly, one study found that diaphragm muscle echogenicity was increased in patients with severe forms of COVID-19 requiring mechanical ventilation [118]. In patients admitted to the ICU, diaphragm ultrasound was initially studied as a possible marker of inspiratory effort during mechanical ventilation, either invasive or non-invasive. However, central venous pressure and esophageal pressure outperformed diaphragm ultrasound for assessing inspiratory effort in that particular setting [119,120]. 

Beyond their pathophysiological significance, diaphragm thickness and excursion, measured with ultrasound on hospital admission, were both recognized as able to predict need for ventilatory support, ICU admission and mortality in patients with moderate to severe COVID-19 [121,122,123,124]. In particular, a poor prognosis was associated with reduced excursion during spontaneous breathing and reduced thickness, but increased thickening fraction, probably as the result of increased muscular workload [125]. 

In the ICU, diaphragmatic dysfunction, defined as a reduced thickening fraction assessed by ultrasound, was predictive of the need for invasive ventilation in patients with severe respiratory failure related to COVID-19 pneumonia undergoing a NIV trial [126,127]. In mechanically ventilated ICU patients, reduced diaphragm thickness was also associated with prolonged ventilation time [128] and mortality [129]. Furthermore, a decrease in diaphragm thickness after five days of ICU stay was recognized as an expression of acute muscle wasting and was significantly associated with mortality [130]. However, the diaphragm thickening fraction was not predictive of the weaning outcome in patients undergoing invasive mechanical ventilation [131]. Instead, ultrasound assessment of diaphragm excursion on spontaneous breathing immediately after weaning of mechanically ventilated COVID-19 patients was able to predict the success of extubation and survival [132,133]. 

Overall, all these studies support the usefulness of diaphragm ultrasound as an aid to clinical decisions and prognosis formulation in patients with severe COVID-19. Unfortunately, none of the studies conducted in this setting were specifically focused on older patients. 

Other investigators have evaluated diaphragm ultrasound after hospital discharge for COVID-19. Diaphragm function is generally not impaired in survivors of moderate forms of COVID-19 pneumonia not requiring ventilatory support [134]. Instead, it is generally impaired in survivors of severe COVID-19 with prolonged ICU stays, but ultrasound was not as effective as the measurement of maximal inspiratory pressure and transdiaphragmatic pressure in detecting dysfunction [135,136]. However, some studies suggested that a subset of patients with long COVID or post-COVID syndrome symptoms (approximately 10%) may actually have diaphragm dysfunction with reduced maximal voluntary excursions detectable on ultrasound [137,138,139,140]. Improvements in diaphragm excursion were also seen in long-term follow-up of post-COVID syndrome patients, either spontaneously or after targeted rehabilitation protocols [141,142]. 

The long COVID syndrome is a complex condition affecting >20% of subjects who survived moderate and severe forms of COVID-19, especially in the earlier pandemic waves [143]. This syndrome leads to particularly relevant consequences in older individuals, increasing the burden of frailty and sarcopenia [144,145], and ultimately causing disability and loss of independence [146,147]. Since diaphragm sarcopenia has a potential negative impact on older patients [19], geriatric post-COVID clinics could represent a promising field of application for diaphragm ultrasound, although this has not yet been explored by the existing scientific literature. 

Despite the frequency in the geriatric population, only two studies have evaluated the role of assessing diaphragm function by ultrasound in patients with bacterial pneumonia to date [148,149]. In the earliest one, diaphragm excursion was negatively correlated with the Acute Physiology and Chronic Health Evaluation (APACHE)-II score and predicted the need for mechanical ventilation and mortality in critical patients [148]. In the second one, conducted on a group of 50 patients presenting to the ED with bacterial pneumonia (mean age 78 years old), a reduced diaphragm thickening fraction was an independent predictor of subsequent respiratory failure [149]. Similar results have also been obtained in a pediatric ED population [150]. Therefore, the predictive role of diaphragm ultrasound assessment in community-acquired pneumonia may need further investigation in the future. 

### 4.4. Congestive Heart Failure and Related Conditions 

Chronic heart failure is associated with diaphragm myopathy characterized by depletion of muscular fibers, fibrosis and infiltration of muscular tissue by adipocytes [151,152]. These changes can ultimately lead to diaphragm dysfunction, with reduced excursion and contractility, which can be easily detected by ultrasound [153]. In fact, in chronic heart failure, the right diaphragm even undergoes a change in its position, descending next to the right renal pelvis, and this alteration also has functional consequences in terms of contractile capacity and efficiency [154]. 

These chronic alterations have important clinical implications in acute heart failure. The main findings of the ultrasound studies dealing with this topic are summarized in Table 4. In a recent investigation conducted on 72 patients with acutely decompensated heart failure and in 100 subjects with normal cardiac function, Scarlata et al. showed that diaphragmatic excursion was reduced in cases of heart failure, with a trend towards reduction with increasing New York Heart Association (NYHA) class, and that diaphragm thickness at any pulmonary volume was increased in comparison with controls [155]. Diaphragm thickness may in fact be influenced by adipose infiltration and fibrosis of the muscular tissue [151,152]. However, subjects with acute decompensated heart failure and reduction in diaphragm thickness generally exhibit poorer physical performance and exercise intolerance, representing a subgroup of patients with an unfavorable prognosis [156] affected by respiratory sarcopenia [19]. Diaphragm sarcopenia, defined as reduced end-expiration thickness, and dysfunction, defined as reduced thickening ratio on spontaneous breathing, show a significant correlation with physical performance (6 min walking test) and fatigue (VO_2_ max), independent of left ventricular ejection fraction [157,158,159,160]. Therefore, diaphragm ultrasound can provide important prognostic information in patients with acute heart failure, guiding treatment and rehabilitation options. 

Interestingly, in a study conducted on 98 patients undergoing maintenance hemodialysis for advanced chronic kidney disease, Zheng and colleagues found that diaphragm dysfunction, defined as a reduced thickening ratio on maximal voluntary inspiration, was significantly predictive of major cardiovascular events or all-cause mortality on a 36-month follow-up [161]. Therefore, diaphragm ultrasonography may provide useful clinical and prognostic information in all conditions associated with impaired fluid balance that imply loss of muscle mass and function. 

### 4.5. Other Conditions 

Diaphragm ultrasound is increasingly used for monitoring respiratory function in patients with amyotrophic lateral sclerosis (ALS) [162]. In this condition, diaphragm thickness is reduced on all pulmonary volumes in comparison with healthy controls [163], and correlates with disease staging [164] and with the number of functional motor units assessed by the phrenic nerve motor amplitude [165]. A reduced diaphragm thickening fraction on maximal inspiration seems the best parameter able to identify diaphragm dysfunction in ALS [166,167], while diaphragm excursion is generally of no clinical importance. Recently, the ratio of the thickening fraction between tidal volume and maximal lung capacity has been proposed as a clinical marker guiding the initiation of non-invasive ventilation in ALS patients [168]. 

Diaphragm paralysis, usually involving only one hemicupola, is an uncommon complication of traumatic lesions or proliferative illnesses of the central nervous system or the phrenic nerve. Stroke can also be a cause of diaphragm paralysis, especially when involving the brain stem. Ultrasound assessment of diaphragm motility and thickness can be useful to diagnose this condition and monitor its evolution [169,170,171]. Respiratory symptom severity is generally related to the degree of dysfunction [169,170,171]. 

Recent studies have also shown that diaphragm function may also be impaired in stroke patients that do not have brain stem involvement [172]. In a study conducted on 48 older hemiplegic patients after stroke, diaphragm motion and thickening fraction were extremely reduced in comparison with 20 matched healthy controls, and correlated with the Berg balance scale score [173]. In another recent study, diaphragm ultrasound parameters correlated with indices of respiratory function and with the National Institutes of Health Stroke Scale (NIHSS) [174]. Interestingly, acute stroke patients with dysphagia had worse diaphragm excursions on spontaneous breathing and on voluntary coughing than acute stroke patients without dysphagia [175], so diaphragm ultrasound could provide clinically relevant information for estimating the risk of aspiration [176]. This potential application is of great interest to acute geriatric patients, but larger studies are needed before recommendations on the use of ultrasound in this setting can be made. 

## 5. Diaphragm Ultrasound in Geriatric Patients with Frailty and Sarcopenia

### 5.1. Structural and Functional Changes of the Diaphragm in Sarcopenia 

Aging is associated with ultrastructural changes in the diaphragm muscle even when sarcopenia is not present. Abnormal size and shape of muscular fibers, increased collagen deposition and reduced fiber cross-sectional area have, in fact, been observed with increasing age in autoptic case series [177]. 

In studies conducted in mice, the presence of sarcopenia was associated with an accelerated decline in age-related reduction in diaphragm fiber cross-sectional area, which was also accompanied by a reduction in maximal specific force, potentially impairing non-ventilatory behaviors essential for adequate airway clearance [178]. These changes did not affect, however, fatigue resistance, and were independent of sex [179]. Fiber clustering was also different in mouse models of sarcopenia, with type I fibers showing a decrease in reciprocal distance, and type II fibers showing an increase in comparison with non-sarcopenic mice [180]. Diaphragm fiber atrophy related to sarcopenia seems to involve almost exclusively type IIx or IIb fibers, which are generally responsible for the generation of higher forces, and not type IIa or type I fibers, resulting in a reduction of diaphragm excursion on maximal voluntary inspiration and in reduced efficiency of cough and other non-ventilatory movements [181]. Conversely, diaphragm muscle fatigability seems not to be significantly affected by sarcopenia, at least in animal models [182]. Furthermore, the diaphragm functional performance was not significantly influenced by extreme ages in older sarcopenic mice, suggesting that sarcopenia could involve respiratory muscles with a threshold effect [183]. 

### 5.2. Diaphragm Ultrasound Studies in Sarcopenic Patients 

In the current state-of-the-art of the scientific literature, very few studies have combined diaphragm ultrasound with a formal assessment of sarcopenia in older subjects. In the ICU setting, diaphragm dysfunction, assessed by ultrasound, is very frequently associated with reduced lower limb strength and muscle size, also assessed by ultrasound, two proxies of acute sarcopenia. Overall, these studies indicate that the association between diaphragm dysfunction and lower limb muscle wasting is predictive of adverse outcomes [130,184,185,186,187,188]. However, these studies were mainly focused on children or adult patients.

Two studies combined ultrasound evaluation of diaphragm and quadriceps muscles in patients with chronic respiratory illness [189,190]. In 40 patients with COPD, quadriceps thickness and its variations during voluntary contractions were significantly correlated with the diaphragm thickening fraction [189]. Moreover, in 16 patients with systemic sclerosis, quadriceps thickness was correlated with diaphragm excursion [190]. 

Only three studies evaluated the associations of diaphragm ultrasound parameters with sarcopenia in geriatric patients [191,192,193]. Their results are summarized in Table 5, and suggest that diaphragm excursion and thickness are reduced in patients with sarcopenia. However, the studies suffer from several limitations, including the relatively low age range of participants, the reduced sample size, the lack of comprehensive ultrasonographic evaluation of both excursion and thickness in the same subjects, and the methodology of sarcopenia assessment, with just one study adopting EWGSOP criteria [192]. Furthermore, the association between diaphragm ultrasound parameters with BMI was not assessed [194]. 

Interestingly, a recent study conducted on 142 adult subjects, either healthy or candidates for lung resection for cancer, suggested that the diaphragm thickening fraction during spontaneous breathing, assessed by ultrasound, was associated with balance [195]. Balance impairment has been recognized as an important part of the physical frailty and sarcopenia syndrome in older patients [196,197]. Furthermore, reduced diaphragm thickness has been recognized as a marker of muscle wasting related to malnutrition in children [198]. Therefore, future studies assessing the relationship between diaphragm dysfunction and sarcopenia in older individuals should also account for balance and nutritional status. 

## 6. Possible Use of Diaphragm Ultrasound in Different Clinical Settings

The analysis of the available scientific literature, summarized in the previous sections, suggests that the clinical significance of diaphragm ultrasound may change according to the characteristics of the patient and the setting in which the exam is performed. The measurement of ultrasound-derived parameters (i.e., excursion on both quiet and deep breathing, thickness on end-expiration, quiet and deep inspiration, thickening fraction) may provide different information according to the clinical picture of the patient. In any case, this information has generally no diagnostic value, but may be helpful to establish prognosis and predict clinical outcomes. 

In critical patients admitted to the ICU undergoing mechanical ventilation, ultrasound assessment of the diaphragm thickening ratio can be useful as an aid to predict the timing and the success of ventilation weaning (Table 6). The same prognostic information may be provided, in older patients, by assessment of diaphragm excursion, but there is insufficient evidence to recommend such an assessment. The capacity of diaphragm ultrasound parameters to predict other clinical outcomes in ICU patients has been studied only in critical COVID-19 patients, where reduced thickness and excursion, and increased thickening ratio resulting from an increased respiratory workload, may be associated with hospital mortality (Table 6).

Diaphragm ultrasound may be also useful in acute patients outside the ICU, hospitalized in general medical and geriatrics wards (Table 6). In acute congestive heart failure, reduced excursion and thickness identify a patient phenotype with a more severe clinical course, and generally a higher NYHA class in stable conditions. In patients with acute exacerbations of COPD, a reduced excursion and thickening ratio on TLC are probably associated with failure of an NIV trial and prolonged hospital stay. Similarly, reduced excursion and thickening fraction are also associated with poor outcomes in patients with respiratory failure caused by bacterial pneumonia, although studies in this setting are scarce. In patients with COVID-19 pneumonia admitted to general wards, the presence of diaphragm dysfunction is also associated with the need for escalating ventilatory support, and mortality, allowing an early identification of subjects with a high risk of adverse outcomes.

Diaphragm ultrasound can also be applied to stable patients in an outpatient setting, as a proxy of lung function or as a means to predict the onset of complications associated with specific illnesses (Table 6). Reduced maximal diaphragm excursion can be useful to elucidate the pathophysiology of chronic dyspnea in long COVID syndrome, and can help identify the risk of diaphragm paralysis and onset of dysphagia in stroke survivors. Evaluation of diaphragm thickening ratio by ultrasound is also an established means of monitoring the progression of amyotrophic lateral sclerosis. Finally, diaphragm ultrasound could be included in the panel of sarcopenia assessment in older individuals, and, thus, find an application in the context of preventive geriatrics.

## 7. Conclusions and Perspectives

Diaphragm ultrasound has multiple potential fields of application in geriatric medicine, ranging from the ICU and semi-intensive respiratory units to outpatient clinics dealing with the prevention and management of physical frailty and sarcopenia. In older patients with acute respiratory illness, the assessment of diaphragm excursion and thickening fraction can be useful to predict the need for mechanical ventilation, the outcome of weaning while on invasive or non-invasive ventilation, and, ultimately, the prognosis. The earliest waves of the COVID-19 pandemic could have represented the ideal situation for the application of diaphragm ultrasound, especially in older adults who were often treated in a low-resource setting, but the lack of competence in this particular ultrasound technique often prevented its widespread utilization. 

In acute medical and geriatric wards, diaphragm ultrasound could help clinicians to identify patients with respiratory sarcopenia, a condition that can potentially worsen the clinical course of acute respiratory illness. In particular, a reduced diaphragm thickening fraction seems to be associated with a poorer prognosis in patients hospitalized with acute exacerbation of COPD. In patients with acute congestive heart failure, the presence of reduced diaphragm excursion correlates with an increasing NYHA class, while a reduction in diaphragm thickening fraction is usually associated with a more severe clinical phenotype. Diaphragm ultrasound could potentially provide important clinical information also in the assessment of the risk of aspiration in acute and subacute stroke. 

Finally, in older outpatients undergoing comprehensive geriatric assessment, diaphragm ultrasound could represent a diagnostic tool to complete the assessment of physical frailty and sarcopenia. However, further studies should assess how diaphragm ultrasound parameters correlate with physical performance, muscle mass and muscle strength. 

Diaphragm ultrasound should also be integrated with thoracic ultrasonography, in the assessment of both acute and chronic patients, as recently suggested by a position paper of the Research Group on Thoracic Ultrasound in the Older Patient by the Italian Society of Geriatrics and Gerontology [199]. Multi-site ultrasound assessment is generally able to improve the clinical management of geriatric patients, and this is particularly true for the frailer ones, who generally do not take advantage of traditional diagnostic examinations. Research in this particular field of geriatric medicine should, thus, be implemented. 

## Figures and Tables

**Table 1:** Summary of the normative values of diaphragm ultrasound parameters, according to studies conducted in healthy individuals.

Author, Year [Ref]	Parameter	Normative Values in Males	Normative Values in Females	Ethnicity of Participants
Ueki, 1995 [39]	Thickness at TLC	4.5 ± 0.9 mm	-	Asian
	Thickness at FRC	1.7 ± 0.2 mm	-	
	Thickness at RV	1.6 ± 0.2 mm	-	
Boussuges, 2009 [27]	Excursion on QB	18 ± 4 mm	16 ± 4 mm	Caucasian
	Excursion on DB	75 ± 9 mm	64 ± 10 mm	
Boon, 2013 [42]	Thickness at FRC	3.8 ± 1.5 mm	2.7 ± 1 mm	Caucasian
	Thickening difference on DB	18 ± 5 mm	18 ± 5 mm	
Harper, 2013 [41]	Thickness at TV	3.7 ± 1.4 mm		Caucasian
	Thickness at FRC	3.2 ± 1.4 mm		
	Thickening ratio on QB	1.20 ± 0.15		
Carrillo-Esper, 2016 [43]	Thickness at FRC	1.9 ± 0.4 mm	1.4 ± 0.3 mm	Caucasian
Scarlata, 2019 [24]	Excursion on DB	65 ± 13 mm	55 ± 14 mm	Caucasian
	Thickness at TLC	2.8 ± 0.5 mm	2.4 ± 0.5 mm	
	Thickness at FRC	1.9 ± 0.4 mm	1.7 ± 0.4 mm	
Spiesshoefer, 2020 [25]	Excursion on QB	17 ± 6 mm	15 ± 5 mm	Caucasian
	Excursion on DB	91 ± 19 mm	75 ± 16 mm	
	Thickness at TLC	6.3 ± 1.7 mm	4.7 ± 1.7 mm	
	Thickness at FRC	2.2 ± 0.8 mm	1.8 ± 0.5 mm	
	Thickening ratio on DB	3.03 ± 0.95	2.77 ± 0.83	
Kabil, 2022 [40]	Excursion on QB	24 ± 5 mm	22 ± 5 mm	Arab
	Excursion on DB	57 ± 13 mm	52 ± 12 mm	
	Excursion on QB (over 65)	23 ± 4 mm		
	Excursion on DB (over 65)	61 ± 22 mm		

TLC = total lung capacity, corresponding to maximal voluntary inspiration; FRC = functional residual capacity, corresponding to end-expiration; RV = residual volume, corresponding to forced expiration; QB = quiet breathing; DB = deep breathing; TV = tidal volume, corresponding to inspiration during quiet breathing.

**Table 2:** Studies that have investigated diaphragm ultrasound in older patients with critical illness undergoing ventilatory support, either invasive or non-invasive.

Author, Year [Ref]	Population	Exposure Variable (Ultrasound)	Endpoint Assessed	Main Findings
Huang, 2017 [72]	40 ICU patients aged ≥80 under IV for ≥48 h and meeting the criteria for spontaneous breathing trial	Diaphragm excursion (DD defined as <10.7 mm)	Maintenance of spontaneous breathing for >48 h	Diaphragm excursion ≥ 10.7 mm was predictive of weaning success (AUROC 0.839)
Kocyigit, 2021 [73]	60 patients with COPD and respiratory failure needing NIV support in ED (mean age 70)	Diaphragm thickness (DD defined as thickening fraction <20% during spontaneous breathing)	NIV failure (worsened blood gas analysis, altered mental status, worsening dyspnea, need for IV)	DD predicted NIV failure (sensitivity 84.6%, specificity 91.5%, PPV 73.3%, NPV 95.6%)
Er, 2023 [74]	32 ICU patients aged ≥65 under IV for ≥48 h and meeting the criteria for spontaneous breathing trial	Diaphragm thickness and excursion	Weaning failure (reintubation or mortality within 48 h after extubation)	Diaphragm excursion was the only parameter associated with weaning failure

ICU = intensive care unit, IV = invasive ventilation, DD = diaphragm dysfunction, AUROC = area under the receiver operating characteristic curve, COPD = chronic obstructive pulmonary disease, NIV = non-invasive ventilation, ED = emergency department, PPV = positive predictive value, NPV = negative predictive value.

**Table 3:** Studies that investigated diaphragm ultrasound in patients with acute exacerbations of COPD.

Author, Year [Ref]	Population	Mean Age	Ultrasound Variable of Interest	Main Findings
An, 2022 [106]	55 patients with COPD, either stable or exacerbated	73 ± 8	Thickening fraction and excursion on maximum inspiration	Reduced thickening fraction (AUROC 0.745) and reduced excursion (AUROC 0.721) were able to classify exacerbation status
Lim, 2019 [107]	10 patients with non-critical acute exacerbation of COPD	80 ± 8	Thickening fraction of the right diaphragm on spontaneous breathing	Thickening fraction improved from the acute phase to improvement of symptoms; no variations in excursion
Cammarota, 2019 [108]	21 patients with acute hypercapnic respiratory failure presenting to ED	70–86 (range)	Diaphragm thickness and excursion under NIV	The amplitude of diaphragmatic excursion predicted NIV success (arterial blood pH > 7.35), but not thickness or thickening fraction
Antenora, 2017 [109]	41 patients with acute exacerbation of COPD and acidosis	76	Change in diaphragm thickness under spontaneous breathing (ΔTdi)	ΔTdi correlated with NIV failure, ICU stay and mortality

COPD = chronic obstructive pulmonary disease, AUROC = area under the receiver operating characteristics curve, ED = emergency department, NIV = non-invasive ventilation, ICU = intensive care unit.

**Table 4:** Studies that investigated diaphragm ultrasound in patients with acute decompensated heart failure.

Author, Year [Ref]	Population	Age	Ultrasound Variable of Interest	Main Findings
Yamada, 2016 [157]	40 patients hospitalized with HFpEF	76 ± 12	Diaphragm muscle thickening at end-inspiration (cut-off < 3.9 mm)	Diaphragm dysfunction was associated with inspiratory muscle weakness and shorter 6MWD
Miyagi, 2018 [156]	77 patients hospitalized with heart failure	72 ± 15	Diaphragm muscle thickening at end-inspiration (cut-off < 4 mm)	Diaphragm dysfunction was associated with older age, lower vital capacity, reduced grip strength, reduced inspiratory muscle strength and shorter 6MWD
Kinugasa, 2018 [158]	62 patients hospitalized with heart failure	72 ± 15	Diaphragm muscle thickening at end-inspiration (cut-off < 4 mm)	Diaphragm dysfunction was more prevalent in patients with dynapenia (reduced muscle strength) or sarcopenia (reduced muscle mass and strength)
Spiesshoefer, 2021 [159]	22 patients with HFrEF (A), 8 patients with HFpEF (B), 19 healthy controls (C)	61 ± 13 (A) 68 ± 9 (B) 57 ± 10 (C)	Diaphragm thickening ratio on maximal inspiration	Diaphragmatic dysfunction was equally present in subjects with HFrEF and in subjects with HFpEF
Andriopoulou, 2022 [160]	25 HFpEF patients, 25 matched controls	64 ± 12	Diaphragm excursion during deep breathing	Diaphragm excursion exhibited a strong positive correlation with VO_2_ in both cases and controls
Scarlata, 2024 [155]	72 acutely decompensated heart failure patients (P), 100 healthy volunteers (C)	78 (76–81) (P) 41 (38-44) (C)	Diaphragm thickness on tidal volume and TLC Diaphragm motion during deep breathing	Diaphragm excursion on TLC is reduced in acute heart failure, with an inverse correlation with NYHA class. Diaphragm thickness is increased in comparison with controls

HFpEF = heart failure with preserved ejection fraction; 6MWD = 6-minute walking distance; HFrEF = heart failure with reduced ejection fraction; VO_2_ = peak oxygen uptake; TLC = total lung capacity.

**Table 5:** Studies comparing diaphragm ultrasound parameters in geriatric patients with and without sarcopenia.

Author, Year [Ref]	Population	Diaphragm Ultrasound Parameter of Interest	Method of Sarcopenia Assessment	Main Findings
Zeng, 2021 [191]	64 older (age ≥ 60) patients undergoing outpatient evaluation for lung cancer or nodules	Diaphragm excursion	ASM/height based on BIA with internally validated cut-offs	Diaphragm excursion on forced deep breathing ≤ 5.27 cm was associated with increased odds of sarcopenia (AUROC 0.778)
Deniz, 2021 [192]	30 sarcopenic and 30 non-sarcopenic subjects (age ≥ 65)	Diaphragm thickness	EWGSOP criteria	Diaphragm thickness was reduced at all pulmonary volumes in subjects with sarcopenia and was an independent predictor of the sarcopenic status
Lee, 2023 [193]	45 healthy volunteers aged ≥65	Diaphragm thickness	ASM/BMI based on BIA measurement	ASM/BMI showed significant positive correlation with diaphragm thickness (r = 0.319). ASM/BMI and diaphragm thickness were predictors of maximal expiratory pressure

ASM = appendicular skeletal muscle mass; BIA = bioimpedance analysis; AUROC = area under the receiver operating characteristic curve; EWGSOP = European Working Group on Sarcopenia in Older People; BMI = body mass index.

**Table 6:** Overview of the main findings of the literature review, in relation to the setting, clinical scenarios and significance of application of diaphragm ultrasound.

Setting	Condition	Ultrasound Parameters of Interest	Clinical Significance
Intensive care unit	Respiratory failure	Reduced thickening ratio (in adults) Reduced excursion (in older subjects)	Prediction of IV weaning failure
Acute-care wards	Exacerbation of COPD	Reduced excursion and thickening ratio on TLC	Prediction of NIV trial failure and duration of hospital stay
	Congestive heart failure	Reduced excursion and thickness	Associated with increasing NYHA class and exercise intolerance
	Bacterial pneumonia	Reduced excursion and thickening fraction	Prediction of progression to respiratory failure, need for IV and mortality
	Viral pneumonia (COVID-19)	Reduced excursion and thickness, increased thickening ratio	Prediction of need for NIV, IV, ICU admission and mortality
Long-term care/outpatient clinics	COPD	Excursion on different pulmonary volumes	Association with spirometric parameters
	Long COVID syndrome	Reduced maximal excursion	Associated with exhaustion and subjective dyspnea (diagnostic aid)
	Amyotrophic lateral sclerosis	Reduced thickening ratio	Marker of disease progression to end-stage respiratory failure
	Previous stroke	Reduced excursion	Associated with diaphragm paresis, dysphagia and reduced balance
	Physical frailty and sarcopenia	Reduced thickness and thickening ratio, reduced excursion	Marker of respiratory involvement of the sarcopenia syndrome, marker of severity

IV = invasive ventilation; COPD = chronic obstructive pulmonary disease; TLC = total lung capacity; NIV = non-invasive ventilation; NYHA = New York Heart Association; ICU = intensive care unit.

## Data Availability

No human data are associated with this article.
